# Cell-Free Fat Extract for the Treatment of Lumbar Disc Degeneration: A Novel Approach Using Adipose-Derived Biologic

**DOI:** 10.3390/biomedicines13061344

**Published:** 2025-05-30

**Authors:** Chenyang Xu, Xianhao Zhou, Cheng Yang, Fanshangmin Zhou, Youzhuan Xie

**Affiliations:** Shanghai Key Laboratory of Orthopaedic Implant, Department of Orthopaedic Surgery, Ninth People’s Hospital, School of Medicine, Shanghai Jiaotong University, Shanghai 200011, China

**Keywords:** IVDD, ferroptosis, inflammation, CEFFE

## Abstract

**Background**: Intervertebral disc degeneration (IVDD) is a major cause of chronic back pain. Recent studies suggest that ferroptosis, a form of cell death, contributes to the degeneration of nucleus pulposus cells (NPCs). This study explores a novel therapeutic strategy using cell-free fat extract (CEFFE), rich in cytokines, to mitigate IVDD by inhibiting oxidative stress-induced ferroptosis. **Methods**: In vitro, NPC degeneration was induced by TNF-α/TBHP. The effects of CEFFE on matrix metabolism were evaluated using Western blotting, RT-qPCR, and high-density culture, with regenerative effects measured via CCK-8 assays. Ferroptosis was assessed by Western blotting, immunofluorescence, and electron microscopy. In vivo, rats with caudal IVDD were treated with CEFFE for 4 weeks, and therapeutic efficacy was evaluated through imaging and histological analysis. **Results**: In vitro, CEFFE reduced TNF-α-induced inflammation and promoted matrix synthesis by inhibiting MAPK and NF-κB pathways. It also activated NRF2 to prevent TBHP-induced ferroptosis. In rats, CEFFE facilitated nucleus pulposus repair and significantly slowed disc degeneration. **Conclusions**: CEFFE is a promising strategy to delay IVDD progression by inhibiting ferroptosis, offering potential therapeutic benefits for disc degeneration.

## 1. Introduction

Low back pain represents a significant worldwide health and socioeconomic challenge, frequently linked to Intervertebral Disc Degeneration (IVDD) [1,2]. Presently, management strategies for low back pain encompass non-invasive approaches, such as physical activity and psychological interventions, alongside pharmacotherapies like non-steroidal anti-inflammatory drugs [3,4]. Although these therapies can provide symptomatic relief, they fail to address underlying tissue damage or impede the progression of disc degeneration. For more severe cases of IVDD, lumbar fusion surgery is typically recommended. However, this intervention may limit lumbar flexibility and negatively impact the patient’s quality of life. Consequently, our research prioritizes addressing early-stage IVDD.

The intervertebral disc, a resilient connector between adjacent vertebrae, comprises three key components: the nucleus pulposus, the annulus fibrosus, and the cartilaginous endplate. The nucleus pulposus functions like a cushion, distributing loads evenly across adjacent structures, whereas the annulus fibrosus handles tension during spinal movements such as bending and straightening [5]. Over time and with degeneration, the nucleus pulposus loses its water content, diminishing its resilience. Simultaneously, the annulus fibrosus becomes compromised, with its structural integrity weakening and fissures forming in the cartilaginous endplates. These alterations are influenced by a range of pathophysiological processes. The extracellular matrix (ECM) within the nucleus pulposus is primarily made up of type II collagen and proteoglycans. In degenerative discs, type II collagen progressively transforms into type I collagen. Moreover, catabolic enzymes of the ECM, including matrix metalloproteinases (MMPs) and a disintegrin and metalloproteinases with thrombospondin motifs (ADAMTs), exhibit elevated expression levels, resulting in ECM breakdown [6,7,8]. The activation of diverse signaling cascades, such as those involving MAPK and NF-κB, is also pivotal for the advancement of IVDD. These pathways facilitate cellular apoptosis, hinder cell proliferation, and intensify inflammatory reactions [9,10,11,12].

Ferroptosis, a key mode of programmed cell demise orchestrated by iron-dependent phospholipid oxidation and modulated by diverse metabolic pathways [13,14], has been implicated in numerous organ injuries and degenerative conditions [15,16,17,18]. Prior research underscores the pivotal role of NPC ferroptosis in the advancement of IVDD [19,20,21,22,23]. As such, inhibiting ferroptosis emerges as a promising therapeutic avenue for addressing IVDD [24].

Recent clinical trials and foundational research have highlighted the therapeutic potential of mesenchymal stem cells (MSCs) and adipose-derived stem cells (ADSCs) in addressing ferroptosis [25,26]. ADSCs have attracted considerable interest due to their ability to secrete cytokines with potent anti-inflammatory and antioxidant effects [27,28]. Nonetheless, the use of exogenous stem cells in clinical settings faces limitations related to immunogenicity and the risk of tumorigenesis [29]. CEFFE is a fluid formulation extracted from adipocytes, which is abundant in a variety of cytokines such as insulin-like growth factor-1 (IGF-1), transforming growth factor-beta (TGF-β), and vascular endothelial growth factor (VEGF). Prior investigations have indicated that CEFFE exhibits anti-apoptotic, antioxidant, and pro-proliferative characteristics [30,31,32,33]. Drawing from these insights, we propose that CEFFE may exert therapeutic benefits for IVDD.

This research seeks to confirm the healing impacts of CEFFE on NPC deterioration through an in vitro model triggered by TNF-α stimulation. Moreover, we delve into the foundational processes and corroborate CEFFE’s effectiveness in a rat model of IVDD.

## 2. Materials and Methods

### 2.1. Preparation of CEFFE

This research involving human adipose tissue was approved by the Ethics Committee of the Ninth People’s Hospital Affiliated with the Shanghai Jiao Tong University School of Medicine (SH9H-2024-T332-1) in 1 September 2024.

The CEFFE provided by the SEME CELL Co. Ltd. (https://semelife.com.cn/, Shanghai, China) was extracted as described previously [34]. Adipose tissues were washed with physiological saline to eliminate any blood and residual tissue fragments. Centrifugation at 1200× *g* for 3 min resulted in the formation of three distinct layers. The top oil layer and the bottom aqueous layer were discarded, leaving only the middle fat layer, which was preserved. This layer underwent mechanical emulsification by being alternately shifted between two 10 mL syringes connected via a three-way stopcock with a 2 mm internal diameter, repeated 30 times. Following this process, the emulsified sample was centrifuged again at 1200× *g* for 5 min, yielding four distinct layers. The third liquid layer was collected for further use. The quality control of CEFFE encompasses the following aspects: (1) Sterility requirements. CEFFE must be prepared under strict aseptic conditions. After collecting the third-layer liquid, the solution is filtered through a 0.22 μm filter membrane to remove potential bacteria and impurities. Endotoxin levels in CEFFE samples are assessed using the rapid gelation method. CEFFE samples must exhibit no detectable endotoxin. (2) Content requirements. CEFFE protein concentration is determined using a BCA Protein Assay Kit (Beyotime, Shanghai, China). The standardized CEFFE protein concentration should range between 2000 and 2500 μg/mL. (3) Packaging and storage. The prepared CEFFE solution is aliquoted into 0.5 mL EP tubes and sealed with parafilm. Samples are stored at −80 °C with a maximum storage duration of 6 months. CEFFE should be immediately used after thawing to avoid repeated freeze/thaw cycles.

### 2.2. Rat Primary Nucleus Pulposus Isolation

Male Sprague Dawley (SD) rats (6–8 weeks old, 300 g, purchased from Shanghai Rat & Mouse Biotech Co, Ltd, Shanghai, China.) were humanely euthanized via intraperitoneal administration of sodium pentobarbital (50 mg/kg body weight). Thereafter, nucleus pulposus tissues were meticulously extracted from the caudal intervertebral discs (Co1-Co6). These tissues underwent digestion using type II collagenase (2 mg/mL) for a period of two hours within a CO_2_ incubator maintained at 37 °C. Post-digestion, the cells were subjected to centrifugation and subsequently resuspended in DMEM medium enriched with 10% fetal bovine serum (FBS) prior to being cultured for further experimental use.

### 2.3. Cell Proliferation Assay

NPCs were inoculated into a 96-well plate at a density of 1500 cells per well. These cells were then exposed to varying concentrations of CEFFE: 0 μg/mL, 50 μg/mL, 100 μg/mL, 200 μg/mL, and 400 μg/mL, either alone or with TBHP. After 24 h of incubation, the wells received a new culture medium supplemented with 10% CCK-8 solution (APExBIO, Houston, TX, USA) and were further incubated at 37 °C for an hour. Optical density readings were taken at 450 nm using a multimode microplate reader (Tecan Life Sciences, Switzerland).

### 2.4. Liperfluo, FerroOrange and ROS Staining

We employed fluorescent probes Liperfluo (Dojindo, Tokyo, Japan), FerroOrange (Dojindo, Japan), and DCFH-DA (Beyotime, China) to investigate ferroptosis within NPCs. NPCs were plated at a concentration of 30,000 cells per confocal dish and given time to attach. The cells were then exposed to TBHP (100 μM) individually or together with CEFFE (200 μg/mL) for 24 h. A 1 mM Liperfluo mixture was created and introduced into the dishes, followed by a 30 min incubation period within a cell culture incubator. Following rinsing with DMEM, fluorescence microscopy was performed utilizing a confocal microscope (Leica, Wetzlar, Germany) with an excitation wavelength of 488 nm and emission detection ranging from 500 to 550 nm.

### 2.5. High-Density Culture

A total of 1.0 × 10^5^ NPCs were suspended in 10 µL of growth medium and dispensed into each well of a 24-well plate. These cells were then incubated at 37 °C for 1 h before an additional 0.5 mL of DMEM medium supplemented with 10 ng/mL insulin–transferrin–selenium (ITS) and 10% fetal bovine serum (FBS) was added to each well. Following another 24 h incubation period at 37 °C, the NPCs were exposed to TNF-α (20 ng/mL) and/or CEFFE (200 μg/mL). The medium was replenished every 2 days. After 7 days of continuous culture at 37 °C, the micro-masses were stained with Alcian blue for 24 h at room temperature (*n* = 3 per group).

### 2.6. RNA Extraction, Reverse Transcription, and RT-qPCR

Total RNA was isolated from the samples using the Axygen Total RNA Extraction Kit. Subsequently, this RNA was converted into cDNA via reverse transcription with PrimeScriptTM RT Master Mix (supplied by Takara, Japan). RT-qPCR was executed on an Applied Biosystems QuantStudio 6 Flex Real-Time PCR System (Thermo Fisher Scientific, Shanghai, China) utilizing Hieff^®^ qPCR SYBR Green Master Mix (Yeasen, Shanghai, China). The PCR primer sequences, crafted through the Pubmed BLAST tool (https://blast.ncbi.nlm.nih.gov/Blast.cgi, 01/05/2024), are detailed in Table S1. Expression levels of target genes were standardized, and the data were processed using the 2^–ΔΔCt^ technique. Statistical evaluation was carried out employing one-way ANOVA.

### 2.7. Extraction of Total Cellular Protein and Western Blot Analysis

Cell proteins were extracted utilizing RIPA buffer augmented with protease and phosphatase inhibitors (APExBIO, USA). Protein concentrations were quantified using the BCA assay kit (Beyotime, Shanghai, China). Equivalent quantities of protein lysates were fractionated on 4–20% gradient SDS-PAGE gels (Genscript, Nanjing, China) and electrotransferred onto PVDF membranes (Merck Millipore, USA). The membranes were prepped with blocking solution containing skim milk for an hour at room temperature, then probed overnight at 4 °C with primary antibodies diluted 1:1000. Antibodies used included β-actin (3700S, Cell Signaling Technology, Shanghai, China), P65 (8242S, Cell Signaling Technology, Shanghai, China), P38 (8690S, Cell Signaling Technology, Shanghai, China), JNK (67096S, Cell Signaling Technology, Shanghai, China), phosphorylated-P65 (3033S, Cell Signaling Technology, Shanghai, China), phosphorylated-P38 (4511S, Cell Signaling Technology, Shanghai, China), phosphorylated-JNK (4668S, Cell Signaling Technology, Shanghai, China), MMP3 (17873-1-AP, ProteinTech, Shanghai, China), MMP13 (18165–1-AP, ProteinTech, Shanghai, China), NRF2 (abs130481, Absin, Shanghai, China), KEAP1 (abs115782, Absin, Shanghai, China), ACSL4 (22401-1-AP, ProteinTech, Shanghai, China), and GPX4 (ab125066, Abcam, Cambridge, UK). After the primary antibody incubation, the membranes underwent washing with TBST. They were then subjected to a 1 h incubation in the dark at room temperature with secondary antibodies: anti-rabbit or anti-mouse IgG. Following three wash cycles, visualization of the protein bands was achieved using an LI-COR Odyssey Fluorescence Imaging System (LI-COR Biosciences, Lincoln, NE, USA).

### 2.8. Transmission Electron Microscopy

To investigate morphological changes, NPCs (*n* = 3/group) were subjected to TBHP exposure (100 μM) for 12 h, followed by pre-exposure with or without CEFFE for another 12 h. After treatment, cells were separated, then fixed overnight at 4 °C in 2.5% chilled glutaraldehyde. Subsequent fixation was carried out using osmium tetroxide. Cells underwent dehydration through a sequential alcohol gradient, followed by washing with propylene oxide and embedding in epoxy resin. Negative staining utilized uranyl acetate and lead citrate. Electron micrographs were acquired using a Hitachi transmission electron microscope (TEM) under these settings: HC/HR select = HC-1, accelerating voltage = 80 kV, emission = 10.2, vacuum = 5.6 × 10^-5^ Pa, adhering to th-e manufacturer’s guidelines.

### 2.9. Immunofluorescence Staining

For the immunofluorescence procedure, NPCs were cultured on cell-climbing slices placed within 6-well plates at a concentration of 2 × 10^5^ cells per well. After a 24 h incubation period, the cells were stabilized using 4% paraformaldehyde in PBS. They were then exposed to primary antibodies—anti-MMP13 (1:200; 18165–1-AP, ProteinTech, Shanghai, China) and anti-p-p65 (1:200; 3033S, Cell Signaling Technology, Shanghai, China)—overnight at 4 °C. Next, the cells were treated with an Alexa Fluor 555-conjugated rabbit secondary antibody (Cell Signaling Technology, Boston, MA, USA) for 1 h at room temperature. Finally, the nuclei were stained with DAPI (1:1000, Beyotime, Shanghai, China).

### 2.10. Animals and Surgery Procedure

The animal experiments were approved by the Animal Care and Experiment Committee of Shanghai Jiao Tong University School of Medicine (SH9H-2023-A778-SB) in 2023. All procedures involving animals were executed in strict alignment with the protocols established by the Animal Care and Experiment Committee of Shanghai Jiao Tong University School of Medicine. The work has been reported in line with the ARRIVE guidelines 2.0.

Four adult male Sprague Dawley (SD) rats (8 weeks old, 300 g) were purchased from Shanghai Rat & Mouse Biotech Co, Ltd. (Shanghai, China). All rats were housed in a barrier environment maintained at a temperature of 20–25 °C and humidity of 40–70%, with a 12 h light/dark cycle. The rats had free access to water and food (SYXK-2021–0007, Shanghai, China). Rats were administered pentobarbital sodium (5 mg/100 g body weight) through intraperitoneal injection for anesthesia. They were then placed in a supine position on a surgical table, and their tails underwent sequential sterilization using saline, iodine solution, and 75% alcohol. Access to the caudal intervertebral discs was achieved, with Co3/4 designated as the sham surgery group and Co4/5 and Co5/6 as the experimental groups. A sterile 20-gauge solid needle was inserted vertically into the caudal intervertebral disc and rotated once to damage the nucleus pulposus, thus inducing IVDD. The needle was left in place for one minute.

For the experimental groups, intervertebral disc injections were administered with either PBS or CEFFE, utilizing a 20 μL microinjector, both one day and two weeks following surgery. Rats that died due to infection, hemorrhage, or anesthesia during the experiment were excluded. Four weeks later, the rats were humanely euthanized; their tails were then removed and carefully stripped of surrounding soft tissue. Ultimately, the tail vertebrae were preserved in a 4% polyformaldehyde solution. All the animals were reported except one dead during the experiment due to the anesthesia.

### 2.11. Histological and Immunohistochemistry

For histological evaluation, we utilized Safranin O-Fast Green and hematoxylin and eosin staining techniques on paraffin-embedded tissue sections. In the context of immunohistochemistry, intervertebral disc sections were stained with antibodies directed against COL2 (GB11021, Servicebio, Wuhan, China) and GPX4 (GB114327, Servicebio, Wuhan, China). Diaminobenzidine served as the chromogen in immunohistochemical procedures, whereas try-cy3 casein was employed for immunofluorescence staining. Image analysis involved quantifying the integrated optical density of the acquired images using Image J version 1.53 software.

### 2.12. Data Statistical Analysis

All experiments were performed in biological triplicates, and the results are presented as mean ± standard deviation (Mean ± SD). The statistical analysis was performed using one-way analysis of variance (ANOVA) in GraphPad Prism 9.0. Prior to the analysis, the normality of data distribution was rigorously assessed. Post hoc comparisons were subsequently conducted with Tukey’s honestly significant difference (HSD) test to determine specific group differences. In the figures, ns indicates no statistical significance, * denotes *p* < 0.05, ** denotes *p* < 0.01, *** denotes *p* < 0.001, and **** denotes *p* < 0.0001.

## 3. Results

### 3.1. CEFFE Promotes Regeneration and Reduces Matrix Degradation of Rat Primary NPCs

**Figure 1:**
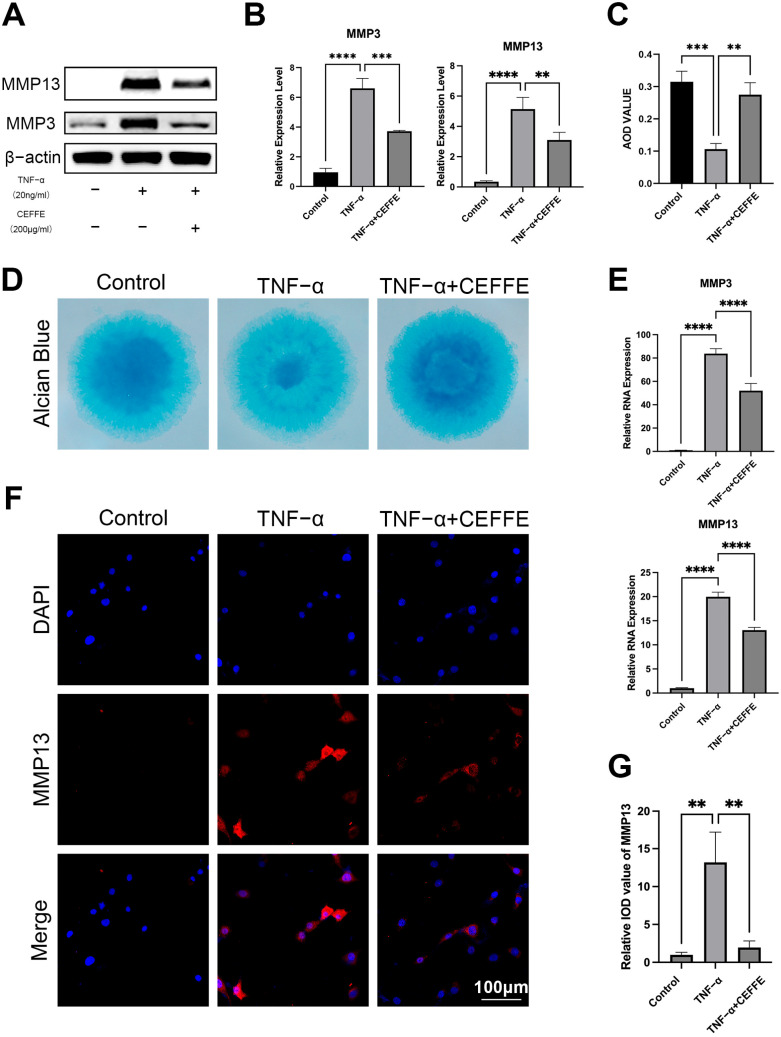
(**A**,**B**) Protein expression of MMP3 and MMP13 in NPCs. CEFFE intervention reduced the TNF-α-induced upregulation of MMP3 and MMP13; *n* = 3. (**C**,**D**) High-density culture of nucleus pulposus cells was stained by Alizarin blue. AOD value was used to evaluate the ECM of NPCs. The difference between each group was analyzed using one-factor analysis of variance, and the results are presented as mean ± standard deviation; *n* = 3. (**E**) mRNA expression of MMP3 and MMP13 in NPCs. CEFFE intervention reduced the expression of MMP3 and MMP13 induced by TNF-α; *n* = 3. (**F**,**G**) Immunofluorescence of MMP13 in NPCs of each group; *n* = 3. ** denotes *p* < 0.01, *** denotes *p* < 0.001, and **** denotes *p* < 0.0001.

NPCs, mirroring the characteristics of chondrocytes, are recognized for their ability to secrete an extracellular matrix (ECM) that is abundant in proteoglycans. To evaluate ECM secretion, we employed Alcian blue staining in high-density cultures of these cells (Figure 1C,D). Our findings showcased a diminished staining intensity in the TNF-α-stimulated group relative to the control group, hinting at reduced ECM secretion and compromised cell vitality under TNF-α exposure. Nonetheless, following treatment with CEFFE, the staining became more pronounced, indicating an increase in ECM secretion.

To explore more deeply how CEFFE influences ECM metabolism, we employed qPCR to investigate the expression levels of critical genes related to ECM metabolism in NPCs, focusing particularly on MMP3 and MMP13 (Figure 1E). After 24 h of exposure to TNF-α, we observed a significant upregulation of MMP3 and MMP13 gene expression, which was effectively counteracted by CEFFE treatment. This observation was supported by Western blot analyses (Figure 1A,B). Immunofluorescence staining further revealed that the MMP13 fluorescent signal in the CEFFE-treated group was notably reduced compared to the TNF-α-stimulated group (Figure 1F,G). Collectively, these results suggest that CEFFE can mitigate the TNF-α-induced degradation of ECM in NPCs, potentially safeguarding them from degeneration.

These findings collectively indicate that CEFFE can attenuate TNF-α-induced ECM degradation in NPCs, thereby potentially preventing their degeneration.

### 3.2. CEFFE Alleviates the Inflammatory Response in NPCs by Inhibiting the NF-κB and MAPK Pathways

**Figure 2:**
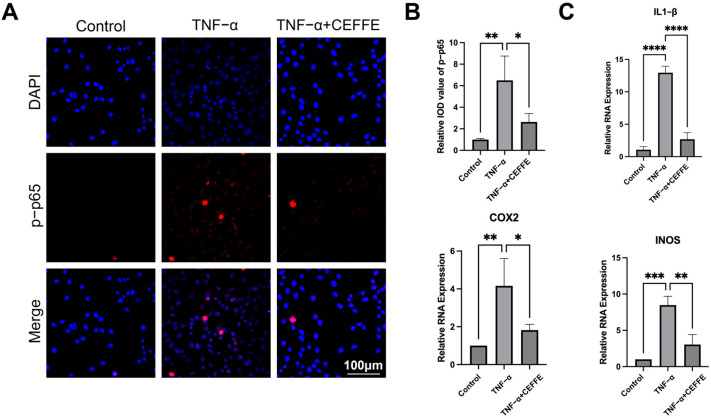
(**A**,**B**) Immunofluorescence showed CEFFE intervention reduced the TNF-α-induced upregulation of p-p65. (**C**) mRNA expression of inflammation in NPCs. After supplementation with CEFFE, COX-2, IL1-β and iNOS decreased; *n* = 3. * denotes *p* < 0.05, ** denotes *p* < 0.01, *** denotes *p* < 0.001, and **** denotes *p* < 0.0001.

To evaluate how CEFFE affects the inflammatory response in NPCs, we employed qPCR to examine pivotal genes associated with inflammation, namely IL1-β, COX2, and iNOS (as shown in Figure 2C). After 24 h of exposure to TNF-α, there was a marked upsurge in the expression of these genes. Yet, administering CEFFE restored the normal expression levels of IL1-β, COX2, and iNOS, suggesting its capacity to temper the inflammatory response triggered by TNF-α.

**Figure 3:**
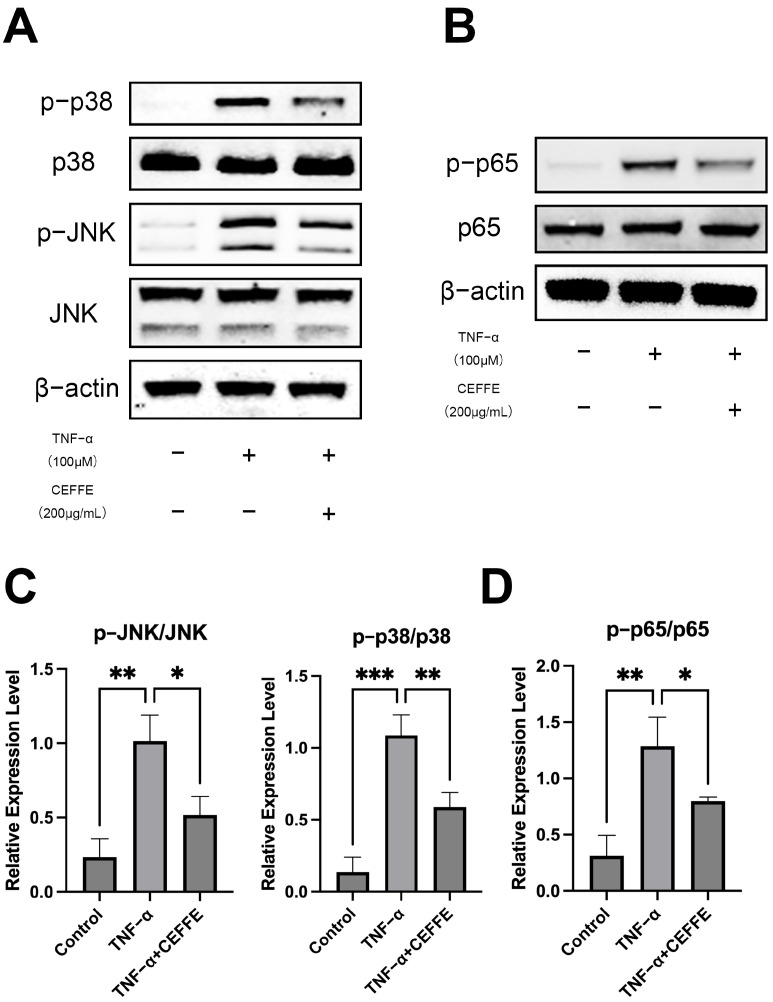
(**A**–**D**) CEFFE attenuates the activation of MAPK and NF-κB signaling pathways. CEFFE intervention reduced the TNF-α-induced upregulation of p-JNK, p-p65, and p-p38 in WB. Immunofluorescence showed the same trend of p-p65; *n* = 3. * denotes *p* < 0.05, ** denotes *p* < 0.01, *** denotes *p* < 0.001.

To gain deeper insights into the anti-inflammatory mechanisms of CEFFE, we utilized Western blotting to assess the activation levels of the MAPK and NF-κB signaling pathways in primary rat NPCs (Figure 3). Following 15 min of exposure to TNF-α, we observed significant phosphorylation of p38, JNK, and p65 proteins, indicating activation of these pathways. Conversely, in cells pretreated with CEFFE, the phosphorylation levels of these proteins were only slightly elevated, implying that CEFFE efficiently mitigates TNF-α-induced activation of the MAPK and NF-κB pathways. Immunofluorescence staining for p-p65 was performed on NPCs under differing conditions (Figure 2A,B). Cells exposed to TNF-α displayed intense fluorescence signals, whereas those treated with CEFFE showed markedly diminished signals relative to the TNF-α-stimulated group. This finding indicates that CEFFE reduces the activation of the NF-κB signaling pathway.

These results underscore the anti-inflammatory properties of CEFFE in NPCs, highlighting its potential therapeutic role in combating inflammation associated with IVDD.

### 3.3. CEFFE Inhibits TBHP-Induced Ferroptosis in NPCs

Expanding upon prior investigations into CEFFE, our study delved into its capacity to alleviate TBHP-induced ferroptosis in NPCs. We concentrated on key features of ferroptosis, including reduced cellular proliferation, the buildup of lipid peroxides and Fe^2+^ ions, heightened reactive oxygen species (ROS) levels, and changes in mitochondrial structure.

**Figure 4:**
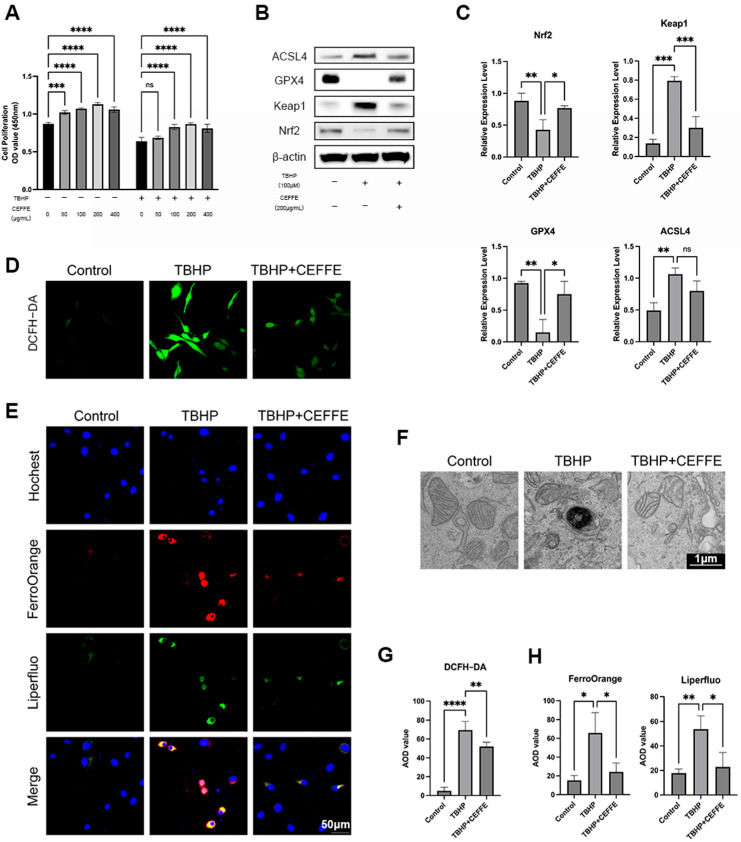
CEFFE inhibits TBHP-induced ferroptosis in NPCs. (**A**) CEFFE promoted cell proliferation of NPCs with or without TBHP in a dose-dependent manner, with the greatest effect observed at a concentration of 200 µg/mL; *n* = 3. (**B**,**C**) Protein expression of NRF2, GPX4, and ACSL4. The expression of GPX4 and NRF2 increased, and the expression of ACSL4 decreased after CEFFE intervention. (**D**,**E**,**G**,**H**) DCFH-DA, FerroOrange and Liperfluo staining of NPCs. After TBHP stimulation, ROS, free Fe2+ and lipid peroxidation increased, while the intervention of CEFFE alleviated this; *n* = 4. (**F**) TEM showed that NPCs stimulated by TBHP exhibited shrunken mitochondria, increased mitochondrial cristae density, and ruptured mitochondrial membranes. After CEFFE intervention, these morphological changes were restored to normal; *n* = 3. * denotes *p* < 0.05, ** denotes *p* < 0.01, *** denotes *p* < 0.001, and **** denotes *p* < 0.0001.

Initially, we explored the impact of CEFFE on cell proliferation in rat primary NPCs across different concentrations (0, 50, 100, 200, 400 µg/mL), both in the presence and absence of TBHP stimulation (Figure 4A). Our findings revealed that CEFFE notably enhanced cell proliferation when TBHP was not present. Upon exposure to TBHP, cell proliferation was suppressed; however, CEFFE efficiently safeguarded the cells against this suppression.

Following TBHP exposure, the concentrations of Fe^2+^ ions, lipid peroxides, and reactive oxygen species (ROS) within NPCs escalated. Nevertheless, supplementing with CEFFE alleviated these rises, as indicated by FerroOrange, Liperfluo (Figure 4E,H), and ROS (Figure 4D,G) fluorescent staining. Transmission electron microscopy further disclosed that TBHP instigated mitochondrial contraction, heightened cristae density, and disrupted outer membranes; however, co-administration of CEFFE restored these mitochondrial structural alterations (Figure 4F).

GPX4, a key modulator of ferroptosis, displayed reduced protein levels after TBHP exposure, an effect that was partly counteracted by CEFFE, as evidenced by Western blot analysis (Figure 4B,C). Moreover, ACSL4—an enzyme involved in lipid metabolism and associated with ferroptosis—showed heightened expression when stimulated by TBHP, an increase that CEFFE helped to mitigate. Nrf-2, a central transcription factor that coordinates antioxidant responses, plays a vital role in activating the body’s defenses against oxidative stress. Protein imprinting studies have shown that CEFFE boosts Nrf2 activation in NPCs, possibly acting as a mechanism to curb ferroptosis.

### 3.4. CEFFE Effectively Prevents IVDD in Rat Model

**Figure 5:**
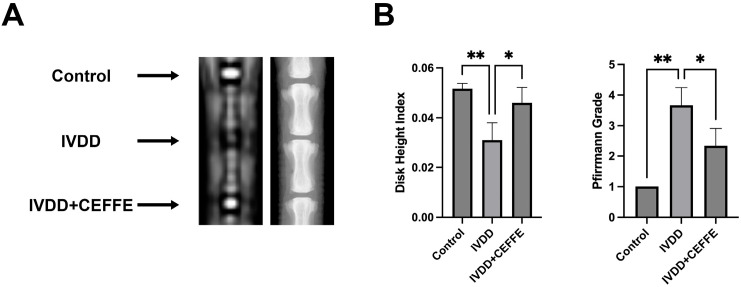
(**A**,**B**) X-ray and MRI of the rat tail vertebrae showed disc space collapse in the puncture group, with reduced signal intensity of the nucleus pulposus on MRI. After CEFFE intervention, DHI and Pfirrmann scores showed significant improvement; *n* = 3. * denotes *p* < 0.05, ** denotes *p* < 0.01.

X-ray and MRI analyses (Figure 5A) disclosed that discs treated with CEFFE displayed a less pronounced decrease in height compared to untreated, degenerated discs. Calculations of the Disc Height Index (DHI) (Figure 5B) corroborated these observations, highlighting the maintenance of disc height in the CEFFE-administered group. Moreover, T2-weighted MRI images demonstrated heightened signal intensity in the CEFFE-treated areas, in stark contrast to the diminished signals seen in untreated, punctured discs. Pfirrmann grading (Figure 5B), employed to evaluate the degree of disc degeneration, reinforced CEFFE’s efficacy in mitigating IVDD.

**Figure 6:**
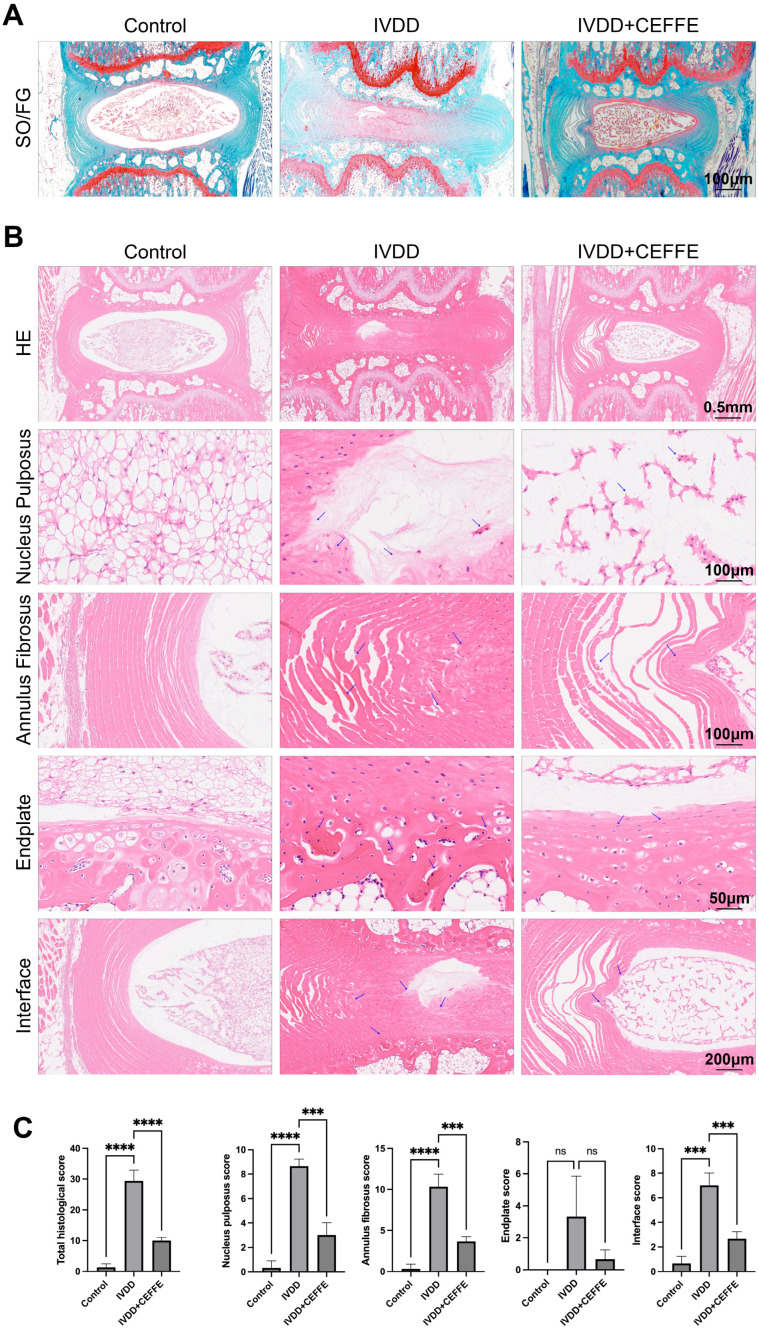
(**A**–**C**) The HE and Safranin O-Fast Green staining for the control group, puncture group, and CEFFE treatment group. After CEFFE treatment, the histological score of the intervertebral disc significantly decreased compared to the puncture group; *n* = 3. *** denotes *p* < 0.001, and **** denotes *p* < 0.0001. The arrows represent typical degenerative changes in intervertebral discs.

**Figure 7:**
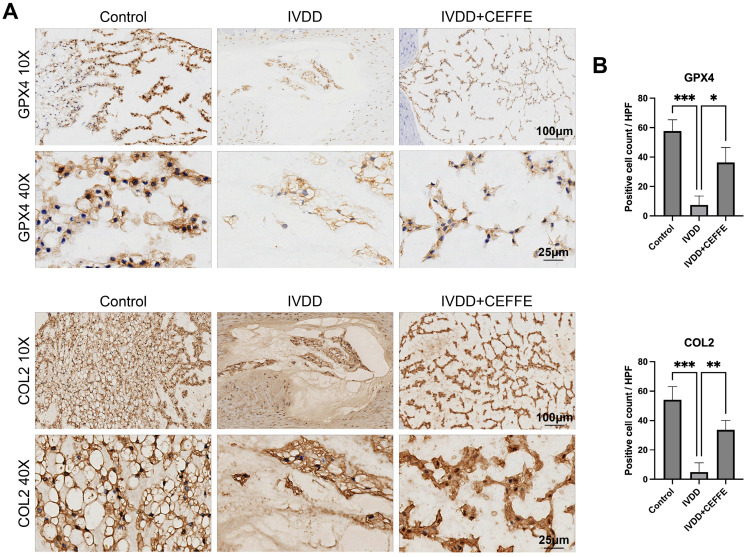
(**A**,**B**) Immunohistochemical staining for COL2 and GPX4 was performed on rat intervertebral disc specimens. The number of positive cells in the nucleus pulposus was quantified using Fiji software. Due to the significant absorption of the nucleus pulposus tissue in the puncture group, only a small number of positive cells can be observed; *n* = 3. * denotes *p* < 0.05, ** denotes *p* < 0.01, *** denotes *p* < 0.001.

Histological assessment via hematoxylin and eosin (HE) staining, employing standardized scoring techniques [35], centered on the integrity of the nucleus pulposus, annulus fibrosus, endplate, and transitional zones. The findings showcased notably higher preservation scores in the CEFFE-treated cohort relative to the IVDD group, signaling milder degenerative changes (Figure 6B,C). Additional Safranin O-Fast Green staining (Figure 6A) revealed a diminished loss of nucleus pulposus material in CEFFE-treated samples compared to untreated IVDD specimens. Immunohistochemical analysis for COL2 and GPX4 (Figure 7A,B) further illuminated the effects of CEFFE on IVDD and ferroptosis within intervertebral discs. The IVDD group showed scanty COL2 and GPX4 expression, typical of degenerated discs, whereas the CEFFE-treated cohort exhibited substantially enhanced protein expression, underscoring CEFFE’s efficacy in maintaining disc integrity and mitigating ferroptotic mechanisms.

These comprehensive findings underscore CEFFE’s therapeutic potential in treating IVDD by preserving disc height, maintaining tissue integrity, and modulating molecular pathways associated with degeneration and oxidative stress.

## 4. Discussion

Current treatments for early IVDD primarily focus on managing symptoms through medication and rehabilitation [3,4], but they do not effectively halt or reverse degenerative changes in the disc. In recent years, biological treatments, including cell therapy, gene therapy, and molecular therapy, have shown promise in clinical trials and animal experiments [36,37]. Stem cell therapy, known for its differentiation potential and secretion of bioactive factors, has garnered attention, yet it carries risks such as tumorigenicity and ethical challenges in clinical application [38]. To address these concerns, cell-free therapies have emerged, with exosomes derived from stem cells serving as viable substitutes [39,40]. CEFFE, containing cytokines similar to those produced by stem cells but devoid of cellular components, offers a less immunogenic alternative [38]. Moreover, adipose tissue, a readily available source often considered clinical waste, provides ample material for producing CEFFE. This study explores CEFFE as a novel therapy for IVDD, confirming its efficacy and investigating the mechanisms through which it alleviates IVDD. By leveraging its bioactive cytokines, CEFFE holds promise in promoting disc regeneration without the drawbacks associated with traditional stem cell therapies.

A key characteristic of IVDD is the metabolic imbalance of the extracellular matrix (ECM) [41]. Type II collagen is the primary protein component of the nucleus pulposus ECM, forming a network structure embedded in proteoglycans. Under stress conditions, ECM remodeling occurs, leading to the conversion of type II collagen to type I collagen and the production of various proteases that promote matrix degradation, such as matrix metalloproteinases (MMPs) and ADAMTs [7,41]. Previous studies have shown that CEFFE contains various growth factors that can promote cartilage regeneration and reduce matrix metalloproteinase levels in osteoarthritis models. This study, through both animal and in vitro experiments, also confirms that CEFFE promotes ECM production and reduces matrix metalloproteinase levels in NPCs.

Extensive research indicates that inflammatory responses promote degenerative changes in nucleus pulposus tissue, and targeting inflammation can significantly alleviate IVDD. Classic inflammatory pathways include the NF-κB and MAPK signaling pathways [12,42,43,44]. Our study shows that CEFFE can inhibit the excessive activation of TNF-α-induced downstream pathways of MAPK, such as JNK, P38, and NF-κB p65. These pathways play crucial roles in cell senescence, apoptosis, and matrix metabolism [12,31,44,45]. However, this study did not focus on the detailed mechanisms of these pathways. Therefore, further in-depth research is needed to explore how CEFFE regulates the downstream inflammatory responses, cell senescence, and apoptosis within these signaling pathways.

Ferroptosis has garnered significant attention from researchers in recent years and has been identified as an important factor in IVDD [14,19,20]. Previous studies have shown that CEFFE can dose-dependently scavenge intracellular ROS and promote the expression of antioxidant enzymes [31,38,46]. However, no studies have specifically focused on the role of CEFFE in ferroptosis. This study investigates the effects of CEFFE on ferroptosis in NPCs through various mechanisms, including scavenging excess ROS, reducing the accumulation of ferrous ions, and eliminating lipid peroxides. The results demonstrate that CEFFE can alleviate ferroptosis in NPCs. Nrf2 is a crucial transcription factor for the oxidative stress response, binding to antioxidant response elements in the promoter regions of many cytoprotective genes. Activation of Nrf2 can prevent cell apoptosis, maintain redox balance, and regulate lipid and carbohydrate metabolism, as well as iron homeostasis, making it a critical regulator of ferroptosis [47]. We found that CEFFE alleviates ferroptosis in NPCs, likely by activating Nrf2.

We used the method reported by Melgoza et al. [35] to evaluate the rat caudal intervertebral disc puncture-induced degeneration model. This method assesses disc degeneration from four aspects: the nucleus pulposus, annulus fibrosus, endplate, and interface. Our findings revealed that in the puncture-induced disc degeneration model, the scores for the nucleus pulposus, annulus fibrosus, and interface were significantly increased. Interestingly, the score for the endplate did not show a significant change compared to the control group. During disc degeneration, fissures often appear in the endplate, leading to Schmorl’s nodes [5]. We believe that the puncture-induced disc degeneration primarily damages the annulus fibrosus and nucleus pulposus tissues, with minimal damage to the endplate. This may be related to the choice of needle thickness. Additionally, the rat caudal vertebrae are less affected by gravity and bear less pressure, which could be another contributing factor.

The rat tail intervertebral disc puncture injury model is a quick, stable, and easy-to-operate model for studying intervertebral disc degeneration. Considering factors such as cost, reproducibility, and modeling time, we chose this model for our study. However, there are some differences between this model and the progression of human intervertebral disc degeneration. Therefore, the results of the animal experiments in this study are only partially indicative, and the effects of CEFFE on human intervertebral discs still require further clinical trials for verification.

This study investigated the therapeutic potential of CEFFE as a novel biotherapeutic approach for intervertebral disc degeneration, with preliminary exploration of its underlying mechanisms. However, the heterogeneous composition of CEFFE precluded precise identification of its bioactive constituents. Notably, unlike stem cell transplantation that sustains paracrine signaling, CEFFE exhibits transient therapeutic effects, while repeated intradiscal administration induced iatrogenic tissue damage. Consequently, future research should prioritize engineered delivery systems (e.g., hydrogel-based sustained-release platforms or minimally invasive microneedles) to address these pharmacokinetic limitations. Another limitation of this study lies in the inherent constraints of the caudal puncture model, which fails to fully recapitulate the clinical progression of intervertebral disc degeneration. Therefore, to address this methodological gap, subsequent investigations will employ alternative animal models, such as surgically induced lumbar instability models in rats. These longitudinal study cohorts will incorporate extended observation periods complemented by systematic behavioral assessments in experimental animals, aiming to refine the translational relevance of preclinical investigations.

## 5. Conclusions

This study demonstrated that CEFFE enhanced cell proliferation, reduced ECM degradation, alleviated inflammation, and inhibited TBHP-induced ferroptosis in NPCs. In conclusion, CEFFE can be a promising strategy to delay the progression of intervertebral disc degeneration in a rat IVDD model.

## Data Availability

The original data can be requested from the corresponding author.

## Supplementary Materials

The following supporting information can be downloaded at: https://www.mdpi.com/article/10.3390/biomedicines13061344/s1, Table S1. Gene primers used in the article.

## Abbreviations

The following abbreviations are used in this manuscript:

ADAMT	A disintegrin and metalloproteinases with thrombospondin motifs
ADSC	Adipose-derived stem cell
CCK-8	Cell counting kit-8
CEFFE	Cell-free fat extract
ECM	Extracellular matrix
FBS	Fetal bovine serum
IGF1	Insulin-like growth factor-1
ITS	Insulin–transferrin–selenium
IVDD	Intervertebral disc degeneration
MMP	Matrix metalloproteinases
NIH	National Institutes of Health
NPC	Nucleus pulposus cell
rt-qPCR	Real-time quantitative polymerase chain reaction
TBHP	Tert-butyl hydroperoxide
TGF-β	Transforming growth factor-beta
VEGF	Vascular endothelial growth factor
