# Supplementary material for: Cell-Free Fat Extract for the Treatment of Lumbar Disc Degeneration: A Novel Approach Using Adipose-Derived Biologic
Source: Biomedicines. 2025 May 30;13(6):1344. doi: 10.3390/biomedicines13061344 (PMC12189628; doi:10.3390/biomedicines13061344)
Supplement: Supplementary file 1 [file biomedicines-13-01344-s001.zip › biomedicines-3532952-supplementary.pdf]

Table S1 Gene primers used in the article.

Rat IL-1β-F	GACTTCACCATGGAACCCGT
Rat IL-1β-R	CAGGGAGGGAAACACACGTT
Rat iNOS-F	GAGTGAGGAGCAGGTTGAGG
Rat iNOS-R	CCAAGGTGTTGCCCTTTTT
Rat COX-2-F	CTCAGCCATGCAGCAAATCC
Rat COX-2-R	GGGTGGGCTTCAGCAGTAAT
Rat MMP-3-F	TGCTCATGAACTTGGCCACT
Rat MMP-3-R	GTGGGAGGTCCATAGAGGGAT
Rat MMP-13-F	CAAGCAGCTCCAAAGGCTAC
Rat MMP-13-R	TGGCTTTTGCCAGTGTAGGT
